# Community organization and network complexity and stability: contrasting strategies of prokaryotic versus eukaryotic microbiomes in the Bohai Sea and Yellow Sea

**DOI:** 10.1128/msphere.00395-24

**Published:** 2024-08-13

**Authors:** Xiaoxiao Wang, Hualong Wang, Yantao Liang, Andrew McMinn, Min Wang

**Affiliations:** 1 College of Marine Life Sciences, Institute of Evolution and Marine Biodiversity, Frontiers Science Center for Deep Ocean Multispheres and Earth System, Key Lab of Polar Oceanography and Global Ocean Change, Ocean University of China, Qingdao, China; 2 UMT-OUC Joint Center for Marine Studies, Qingdao, China; 3 Institute for Marine and Antarctic Studies, University of Tasmania, Hobart, Australia; University of Wisconsin-Madison, Madison, Wisconsin, USA

**Keywords:** network complexity and stability, prokaryotic and eukaryotic plankton, community organization, Bohai Sea and Yellow Sea

## Abstract

Unraveling the effects of spatial gradients on microbiome assembly and association is a challenging topic that remains understudied in the coastal ecosystem. Here, we aimed to investigate the effects of spatial variation on the network complexity and stability of plankton microbiomes in the Bohai Sea and Yellow Sea. These seas serve as spawning and nursery grounds for economically important fisheries valued at billions of dollars annually. Environmental heterogeneity structures microbial communities into distinct spatial patterns, leading to complex direct/indirect relationships and broader ecological niches of bacterioplankton compared to microeukaryotic communities. Interestingly, salinity gradients positively influenced the richness of rare subgroups of bacterioplankton, while the rare microeukaryotic subgroups showed an opposite trend. Abundant subgroups of prokaryotic/eukaryotic microbiomes exhibited greater environmental niche breadth and lower phylogenetic distance compared to the rare subgroups. Stochastic processes contributed greatly to microbiome dynamics, and deterministic processes governed the bacterioplankton organization with a lower phylogenetic turnover rate. Compared to microeukaryotes, bacterioplankton exhibit higher network modularity, complexity, and robustness and lower fragmentation, and vulnerability. These observations offer vital insights into the anti-interference ability and resistance of plankton microbiomes in response to environmental gradients in terms of organization and survival strategy as well as their adaptability to environmental disturbances.

**IMPORTANCE:**

An in-depth understanding of community organization and stability of coastal microbiomes is crucial to determining the sustainability of marine ecosystems, such as the Bohai Sea and Yellow Sea. Distinct responses between prokaryotic and eukaryotic microbiomes to spatial heterogeneity were observed in terms of geographical distribution, phylogenetic distance, niche breadth, and community assembly process. Environmental variations are significantly correlated with the dynamics of rare eukaryotic plankton subcommunities compared to prokaryotic plankton subcommunities. Deterministic processes shaped prokaryotic plankton community organization with a lower phylogenic turnover rate. Rare subgroups had noticeably higher phylogenetic distance and lower niche breadth than the corresponding abundant subgroups. Prokaryotic microbiomes had higher molecular network complexity and stability compared to microeukaryotes. Results presented here show how environmental gradients alter both the geographical characteristics of the microbial organization in coastal seas and also their co-occurrence network complexity and stability and thus have critical implications for nutrient and energy cycling.

## INTRODUCTION

Marine microbiomes play an important role in biogeochemical cycling and account for more than half of global primary production (1, 2). Microbial communities are critical biological drivers in primary production; hence their biomass has a significant influence on trophic web structure and competition for space, nutrients, and minerals (3–5). Clarification of their organization mechanisms is necessary to comprehend how marine planktonic microorganisms contribute to marine ecosystem functioning and their impact on climate processes on larger spatial and temporal scales (2).

Coastal marine ecosystems are strongly affected by anthropogenic activity, such as the Bohai Sea and Yellow Sea. Eutrophication, ocean warming, and other climate changes are likely directly/indirectly affecting microbial dynamics and primary production and also indirectly affecting the Yellow Sea Warm Current (6). The Bohai Sea is a semi-enclosed bay that connects with the more open Yellow Sea. Residence time in the Bohai Sea is longer than that of the Yellow Sea (Bohai Sea, 11–12 years; the Yellow Sea, 5–6 years), where residence time is defined as the amount of water in the region divided by either the source water that enters the sea or the rate of loss from it (7–9). The Bohai Sea and Yellow Sea are important spawning and hatching grounds for many commercial fisheries. The fishing industry in these areas is valued at one billion US dollars per year and includes several important aquatic products, such as chub mackerel, pacific cod, and scaly hairfin anchovy (10–12). Within the Bohai Sea and Yellow Sea coastal ecosystems, anthropogenic impacts are threatening a variety of vulnerable native species causing dramatic alterations to the microbial communities and disrupting the microbial food webs and their ecological functions (13). Community stability is an important measure of the susceptibility of microbial communities to environmental disturbance or anthropogenic pollution (14), where community complexity is characterized by network topological properties such as nodes, edges, average degree, average clustering coefficient, etc. Understanding how the complexity and stability of microbial communities in coastal ecosystems respond to changing environmental conditions such as eutrophication remains challenging (15).

Gradients in nutrient availability and hydrodynamic characteristics significantly shape the microbial community structure, spatial dynamics, and distribution patterns in the Bohai Sea and Yellow Sea (7, 16, 17). Fu et al. (18), for example, showed that the water of the Bohai Sea and the Northern Yellow Sea exhibited distinct physicochemical characteristics attributed to the presence of different water masses and circulation patterns, and these impacted the diversity index of phytoplankton communities, which was higher in autumn and lower in summer (18). Yu et al. (7) found that nitrite and turbidity were the main environmental factors affecting the surface bacterial distribution in the Bohai Sea and Northern Yellow Sea (19). Wang et al. (16) demonstrated that the abundance of phytoplankton in the Bohai Sea was more closely linked to the N:P ratio during summer (16). Wang et al. (5) have also pointed out that bacterial communities were related to the availability of phosphorus and nitrogen in the Bohai Sea (5). However, cross-kingdom comparisons that include both prokaryotic and eukaryotic plankton communities are still rare in coastal ecosystems with most previous studies focusing separately on microeukaryotes or bacteria (13). So far, to our knowledge, a detailed analysis of microbial organization complexity and stability across kingdoms in coastal marine ecosystems, such as the Bohai Sea and Yellow Sea, is still missing.

Network stability is a measure of the resistance of plankton microbiomes to environmental variability and resilience to disturbances ( 14, 20). To understand whether and how spatial gradients affect the organization of marine microbiomes, multiple indices have been applied to characterize the complexity and stability of the microbial community networks and their members, including robustness (21), vulnerability (22), and fragmentation (23). For example, network robustness represents the resistance of a microbial ecosystem to additional extinction after species removal. Several network topological properties such as species richness can estimate community-level complexity (15, 24–26). Deciphering the relationships between microbial complexity and stability could help to understand how microbial diversity and association contribute to the stable supply support of ecological functions. Previous studies on soil microbial communities have found that microbial networks with high complexity exhibit greater stability and robustness and lower vulnerability, indicating increased stability under warming conditions (22, 27–30).

Abundant and rare microbial sub-communities show distinct responses to variations in aquatic environments (4, 31, 32). Rare subgroups are crucial for regulating aquatic environmental functioning and nutrient cycling; they can drive the response of coastal ecosystems to environmental variability and can become dominant under favorable conditions, hence supporting community persistence and stability (4). Rare species can make a substantial contribution to ecosystem function, thus demonstrating the importance of studying inconspicuous species to preserve sustainable ecosystems (13). However, a comprehensive view of the dynamics of abundant/rare microbiome subgroups and microbial community complexity and stability response to spatial heterogeneity in the Bohai Sea and Yellow Sea has not been defined.

In this study, the organization and network complexity and stability of prokaryotic and eukaryotic plankton communities in the Bohai Sea and Yellow Sea along the spatial gradients were systematically researched. A multivariate regression tree (MRT) and piecewise structural equation model (SEM) statistical analyses were applied to determine how marine environmental gradients contribute to shaping the distribution of microbiomes. Niche breadth and a neutral community model were analyzed to identify the assembly process of microbiomes and abundant/rare subgroups in the research area. Community assembly describes how ecological processes at different temporal and spatial scales interact to determine both the species composition and local biodiversity of a microbial community (33). In order to test the stability of co-occurrence prokaryotic and eukaryotic plankton networks, the robustness, vulnerability, and responsiveness of network fragmentation to the removal of significant nodes (i.e., with the highest betweenness centrality) was assessed. The stability of these complex ecological networks and the use of community and environmental data were further analyzed to find whether (i) the microbial community network complexity and stability varied significantly between prokaryotes and microeukaryotes, (ii) the environmental gradients directly/indirectly shaped the organization of the marine microbiomes. The main assumptions of this research state that environmental heterogeneity structures eukaryotic and prokaryotic plankton microbial communities in different ways, and consequently influence their network stability mechanisms. They also assume that clear spatial patterns shape eukaryotic and prokaryotic plankton microbial communities into distinct distribution dynamics, phylogenetic distance, assembly processes, and niche breadth. This study sheds light on the survival and adaptation strategies of eukaryotic and prokaryotic plankton communities in the Bohai Sea and Yellow Sea and improves our base understanding of how cross-kingdom communities maintain the sustainability of coastal microbial ecosystems (34).

## RESULTS

### Diversity and distribution of microbiome and its subgroups

The diversity of prokaryotic and eukaryotic plankton communities was fairly constant along spatial gradients in the Bohai Sea and Yellow Sea (Table S1). In addition, the observed amplicon sequence variant (ASV) richness of microeukaryotic communities was negatively correlated with latitude and positively related to the nitrite gradients in the Bay (*P* = 0.0367, *R*^2^ = 0.3965), whereas bacterioplankton community richness was not (*P* = 0.819, *R*^2^ = −0.045, Table S1). Similarly, Faith’s phylogenetic diversity of microeukaryotic communities increased along the estuarine nitrite gradient while the bacterioplankton community remained rather constant.

The diversity of abundant and rare subgroups of microbiomes showed distinct spatial shifts compared to the total microeukaryotic and bacterioplankton communities, respectively (Table S1). The diversity of abundant microeukaryotic subgroups did not relate to the environmental gradients, except for the observed ASV richness which had a significant relationship with dissolved oxygen. For rare subgroups of eukaryotic plankton, it was noticeable that Pielou’s evenness and the Shannon-Wiener index were strongly correlated with the geospatial gradients (latitude) and the concentration of dissolved oxygen, nitrite, and ammonia.

There were no significant relationships between the diversity indices of abundant prokaryotic plankton subgroups and environmental gradients. The diversity of rare bacterioplankton subgroups was less affected by environmental gradient compared to the microeukaryotic communities. Richness and the Shannon-Wiener index of rare bacterioplankton subgroup were significantly related to salinity and chlorophyll *a* (chl *a*) concentration. Interestingly, the salinity gradients were negatively correlated with the richness of rare bacterioplankton subgroups but positively related to the richness of rare microeukaryotic subcommunities, although the diversity of total eukaryotic and prokaryotic plankton communities was generally uniform. These results show that different subgroups of prokaryotic and eukaryotic plankton communities have distinct responses and survival strategies in response to environmental gradients, such as salinity.

Distribution and abundance of prokaryotic and eukaryotic plankton communities and their abundant and rare subgroups were significantly affected by the environmental variations, especially the geographic shifts (Table 1; Table S2; Fig. S3). Geographic variation strongly shaped the microbial distribution and their subgroups (Table 1; Fig. S4), while the other environmental factors structured the microbial distribution to different degrees (Table 1; Table S2). The spatial gradients and nutrient availability strongly shaped the distribution of abundant subgroups compared to the rare subgroups. For example, the distribution of total communities was significantly related to the salinity gradients, but only the distribution of the abundant group of microeukaryotes was significantly affected by these gradients. The temperature had a significant impact on the distribution of prokaryotic plankton communities, while the dissolved inorganic phosphorus (DIP) concentrations and silicate gradients shaped the dynamic of microeukaryotes. The distribution of microbiomes and abundant subgroups might be the key factors that cause variation in the chl *a* concentration in this region. Similarly, the effects of major environmental factors on microbiome distribution were also demonstrated after categorizing the environmental gradients into multiple groups (Table S2). These results show that environmental heterogeneity structured the prokaryotic and eukaryotic microbiomes and subgroups to different extents.

**TABLE 1:** Mantel test on the relationships between eukaryotic and prokaryotic plankton communities and their subgroups with environmental variables^*a*^

Environmental variable	Prokaryotic plankton			Eukaryotic plankton		
	All	Abundant	Rare	All	Abundant	Rare
Geographic distance	0.3256***	0.2943***	0.3309***	0.4832*	0.4437***	0.224***
Salinity	0.1662***	0.151	0.032	0.1596*	0.1533**	0.04
Temperature	0.1628*	0.206	0.033	0.012	0.241	0.001
DO	−0.012	0.07	−0.075	0.095	0.126	0.056
Chl *a*	0.2054*	0.1775*	0.088	0.1622*	0.1445*	0.055
Total nitrogen	0.1888**	0.192**	0.107	0.005	0.01	−0.006
Inorganic N	0.2364**	0.2345**	0.055	0.1387*	0.1862*	0.1471*
Nitrite	0.2677**	0.2742*	0.059	0.026	−0.02	0.023
Nitrate	0.23537**	0.2371**	0.022	0.1781*	0.1501*	0.1882**
Ammonia	0.102	0.111	0.1349*	0.091	0.075	0.032
DIP	−0.005	0.02	−0.001	0.1621*	0.1809*	0.078
Dissolved reactive silicate	−0.102	−0.066	−0.132	0.109	0.139	0.1474*

^*a*^ Asterisks denote levels of significance (*P < 0.05; **P < 0.01; ***P < 0.001); DO, dissolved oxygen.

### Contribution of environmental heterogeneity to microbiome dynamics

The MRT analysis showed the relationship between microbiome composition and environmental variables in a visualized tree with several splits based on nutrient concentration, temperature, latitude, and salinity gradients (Fig. S5). The prokaryotic plankton communities were split by environmental gradients, including temperature, latitude, oxygen, and salinity. Initially, two samples with temperatures exceeding 27.9°C were separated from the remaining 36 samples with temperatures below 27.9°C. Then, 10 samples were clustered as a separate group with each from a latitude lower than 34°N. Similarly, the eukaryotic plankton community composition was divided by nitrogen, latitude, and temperature. The first two groups contained two samples with nitrogen levels lower than 10.81 mg/L. The numerical ranges of the environmental factors derived from MRT provided supportive information to classify the microbial community composition into different subgroups to better understand the impact of environmental gradients on microbiome distribution in the Bohai Sea and Yellow Sea.

**Fig 1:**
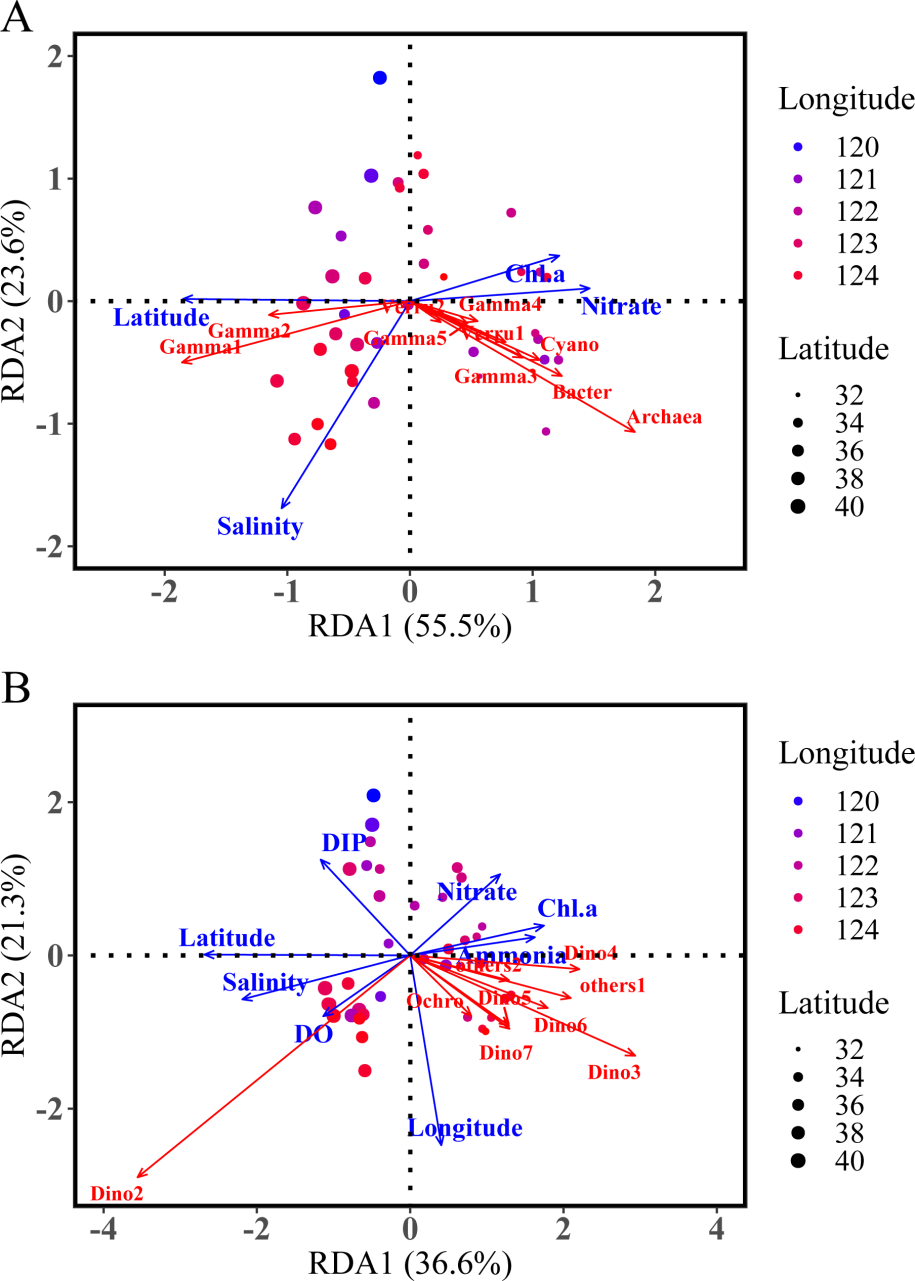
RDA analysis ordination plot indicating patterns of environmental variables influence on eukaryotic (**A**) and prokaryotic (**B**) plankton community composition. The color of the bubble dots represents longitude and size represents latitude. Only significant explanatory environmental factors (*P* < 0.05) (blue) and taxa (ASVs) with goodness-of-fit ≥0.48 for microbial plankton (red) are included in the graph.

The redundancy analysis (RDA) results illustrate that the environmental gradients structure the plankton microbial communities into a clear spatial pattern and explain variations in eukaryotic plankton communities, although to a lesser degree (57.9%) than the prokaryotic plankton communities (79.1%) (Fig. 1). Clearly, microbiome samples collected along the latitude and longitude were separated by RDA1 and RDA2, respectively. In the RDA plot of eukaryotic plankton, the horizontal axis (RDA1) explained 36.6% of the variability with the environmental variables making the largest contributions being latitude, DIP, ammonia, and nitrate concentrations. The vertical axis (RDA2, 21.3% explicability) separated all eukaryotic plankton samples spatially and was mostly explained by DIP, nitrate, and dissolved oxygen (Fig. 1). Similarly, the spatial pattern of prokaryotic plankton was mainly explained by latitude, and nitrate, on the horizontal axis (55.5% explicability) and salinity on the vertical axis (23.6% explicability) (Fig. 1).

**Fig 2:**
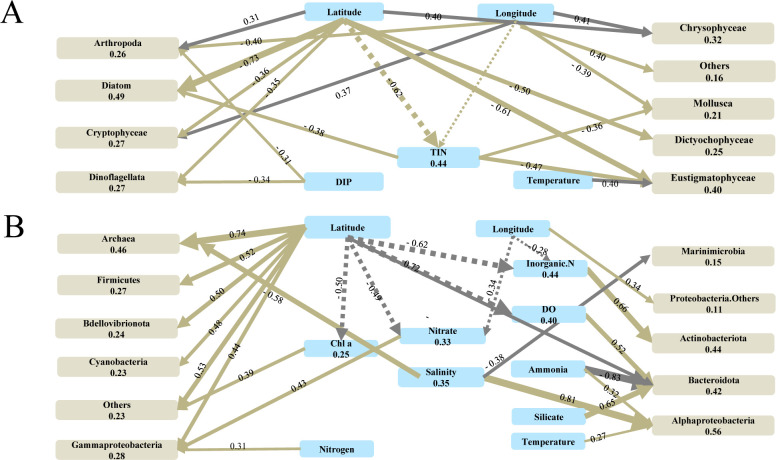
Piecewise SEM to indicate the connection between environmental factors’ influence on dominant eukaryotic (**A**) and prokaryotic (**B**) plankton community distributions. Gray/brown lines indicate significant negative/positive relations between factors or between factors and species. The thickness of the arrows and the number associated with each line represents the strength of correlations. Numbers in parentheses show the explanation degree for each factor.

The distribution of major microbial groups was significantly related to the combinations of explanatory variables (Fig. 1 and 2; Table S1). For example, nitrate, chl *a,* and ammonia gradients were positively correlated with the relative abundance of some Dinophyceae (labeled as Dino 5-7), and Ochrophyta taxa (Eustigmatophyceae), but these species were negatively related to the DIP, dissolved oxygen (DO), salinity, and latitude gradients (Fig. 1). Bacterioplankton dynamics were mainly explained by the environmental factors chl *a*, salinity, latitude, and nitrate. For example, chl *a* and nitrite concentration were positively related to taxa affiliated to Candidatus Actinomarina, Puniceicoccaceae (Verrucomicrobiota), and Gammaproteobacteria, such as UBA10353 marinegroup (Gamma 3), Photobacterium (Gamma 4) and OM182 clade (Gamma 5), while Methylophilaceae (Gamma 1) and, Halieaceae (Gamma 2) were negatively correlated (Fig. 1).

The results of the piecewise SEM analysis demonstrated that environmental parameters were directly/indirectly associated with the prokaryotic plankton variation with more complex relationships than those of the eukaryotic microbial communities (Fig. 2). The results show that latitude, salinity, and nutrient availability together directly influence the distribution and associations of microbiome groups. Additionally, they indirectly affect microbial dynamics by influencing other environmental parameters such as chl *a* and inorganic nitrogen concentration (Fig. 2). For instance, an increase in latitude was associated with decreased diatoms, dinoflagellates, Cryptophyceae, Dictyochophyceae and Eustigmatophyceae, and increased abundance of Chrysophyceae and Arthropoda. Dinoflagellates and diatoms were negatively correlated with phosphate concentration. Increased inorganic nitrogen was associated with decreased diatoms, Mollusca, and Eustigmatophyceae (Fig. 2). Similarly, prokaryotic plankton distributions, such as increased Cyanobacteria, Gammaproteobacteria, Firmicutes, Archaea, and Firmicutes, and decreased Bacteroidota, were significantly linked to latitude. Although there were correlations with nitrate and total inorganic nitrogen, the latitude also affected other microbial groups such as Gammaproteobacteria and Actinobacteria (Fig. 2). The mutual influences between environmental conditions and microbial geographic distribution were demonstrated to be complex in this coastal area.

In general, these results demonstrated how spatial variation in this region is directly and indirectly associated with the community distribution of dominant eukaryotic and prokaryotic plankton groups.

### Phylogenetic distance and niche breadth of plankton microbiome

**Fig 3:**
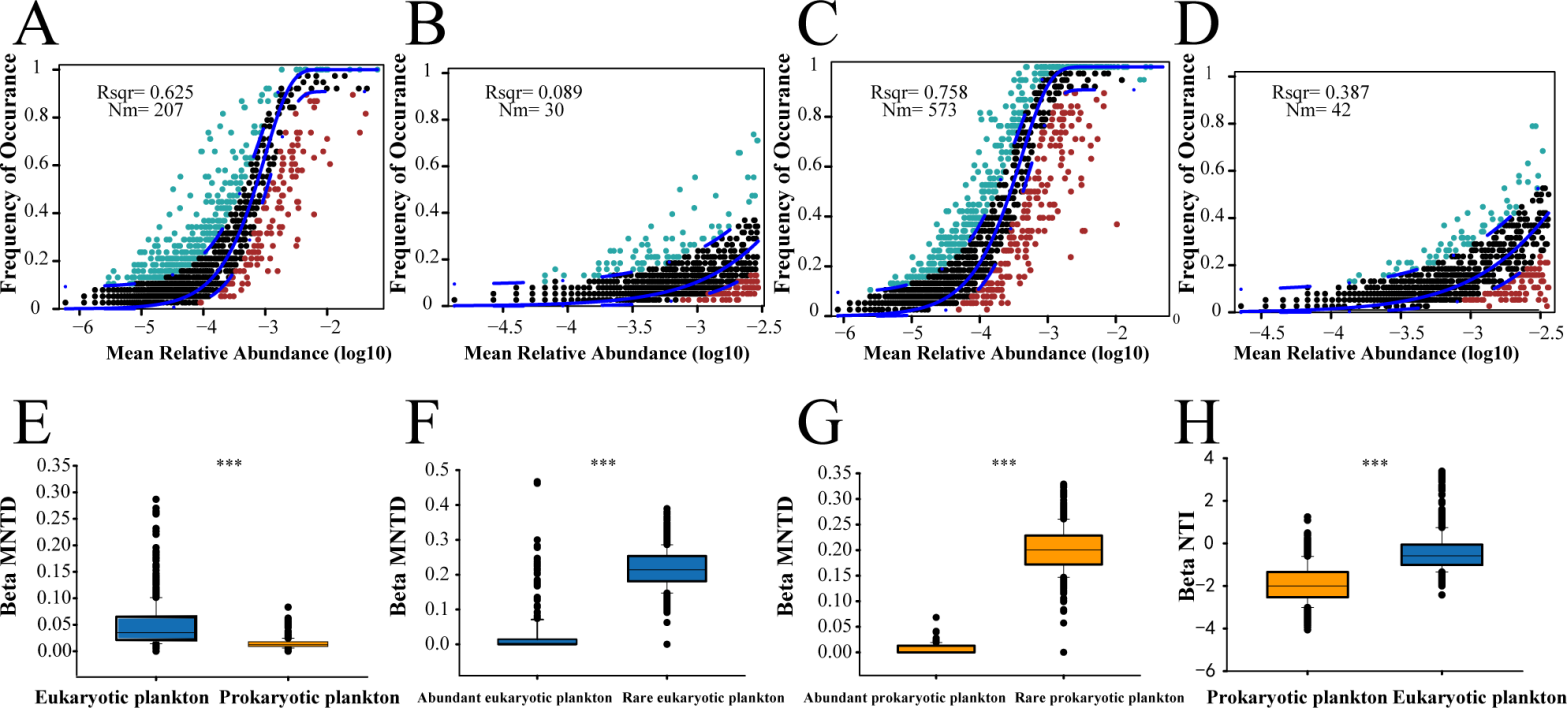
Neutral community model and phylogenetic analysis of prokaryotic and eukaryotic plankton communities. The neutral community model indicates the difference in community distance of (A) total microeukaryotic microbial communities, (**B**) rare microeukaryotic subgroups, (**C**) prokaryotic plankton communities, and (D) rare prokaryotic subgroups. Phylogenetic analysis demonstrates (E) phylogenetic distance based on βMNTD matrix between eukaryotic and prokaryotic plankton communities, (**F**) phylogenetic turnover rate based on β nearest taxon distance (βNTI) matrix between eukaryotic and prokaryotic plankton communities, (**G**) phylogenetic distance based on βMNTD matrix between abundant and rare subgroups for prokaryotic plankton communities, and (H) phylogenetic distance based on βMNTD matrix between abundant and rare subgroups for eukaryotic plankton communities.

Mean nearest taxon distance (SES MNTD), which reflects the phylogenetic evenness characteristics of prokaryotic and eukaryotic microbiomes, showed that species were more distantly related than expected by chance (Fig. S6). β mean nearest taxon distance (βMNTD) statistical analysis results showed that the microeukaryotic community had a higher polygenetic distance than the prokaryotic plankton community (*P* < 0.01) (Fig. 3E). Furthermore, the rare subgroups of prokaryotic and eukaryotic microbiomes had noticeably greater phylogenetic distance compared to the corresponding abundant subcommunities. This suggests a distinct phylogenetic adaptation strategy of the microbiome subgroups as to how they responded to environmental heterogeneity (Fig. 3F and G).

**Fig 4:**
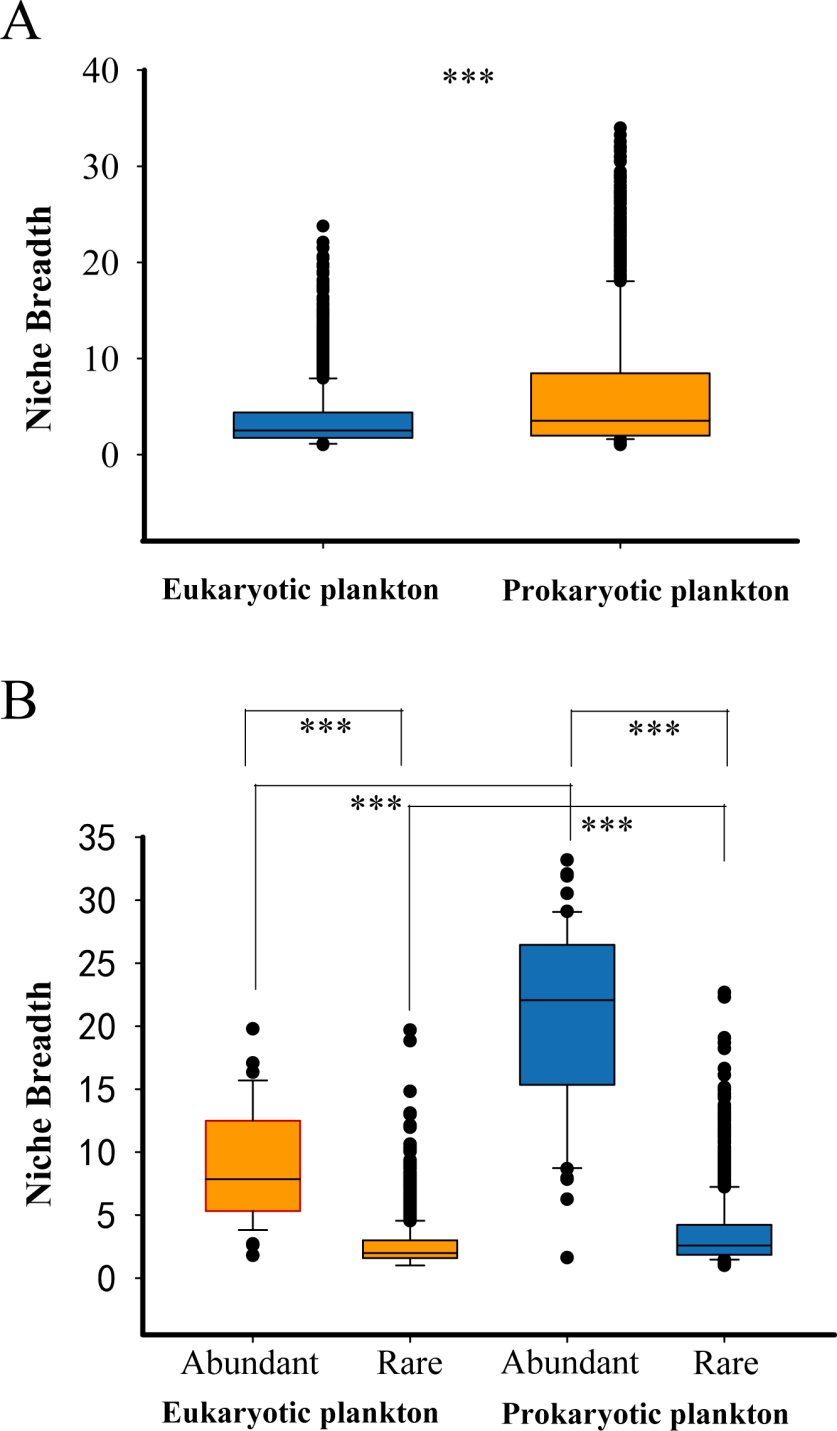
Environmental niche breadth analysis of (A) eukaryotic and prokaryotic plankton communities and (B) abundant and rare subcommunities.

The relative contributions of ecological processes differed between prokaryotic and eukaryotic plankton communities (Fig. 4). Dispersal limitation belonging to stochastic processes dominated in the eukaryotic microbial community (|β NTI| < 2) (Fig. 3H). Deterministic processes governed the organization of prokaryotic plankton communities with a lower phylogenetic turnover rate (β NTI < −2) (Fig. 3H).

Prokaryotic plankton communities exhibited greater environmental breadth than eukaryotic plankton communities (Fig. 4A) and their abundant subgroups had a comparatively broader environmental breadth than the rare subgroups (Fig. 4B). In addition, abundant/rare prokaryotic subgroups showed a greater environmental breadth than abundant/rare eukaryotic subgroups, respectively.

### Neutral community models of plankton microbiomes

Neutral community models of prokaryotic and eukaryotic plankton communities both had a high explanation rate and species diffusion, respectively (*R*^2^ = 0.758, Nm = 573 for prokaryotic microbial communities, *R*^2^ = 0.625, Nm = 207 for eukaryotic microbial communities). This indicated that stochastic processes were critical for the organization of both prokaryotic and eukaryotic microbial communities (Fig. 3A and C). However, the rare subgroups were less affected by a stochastic process with a lower explanation rate and species diffusion degree compared with the total microbial community (Fig. 3B and D). Overall, stochastic processes made a strong contribution to microbiome dynamics, while the assembly of rare subgroups was mainly affected by environmental gradients.

### Community complexity and stability of plankton microbiomes

**Fig 5:**
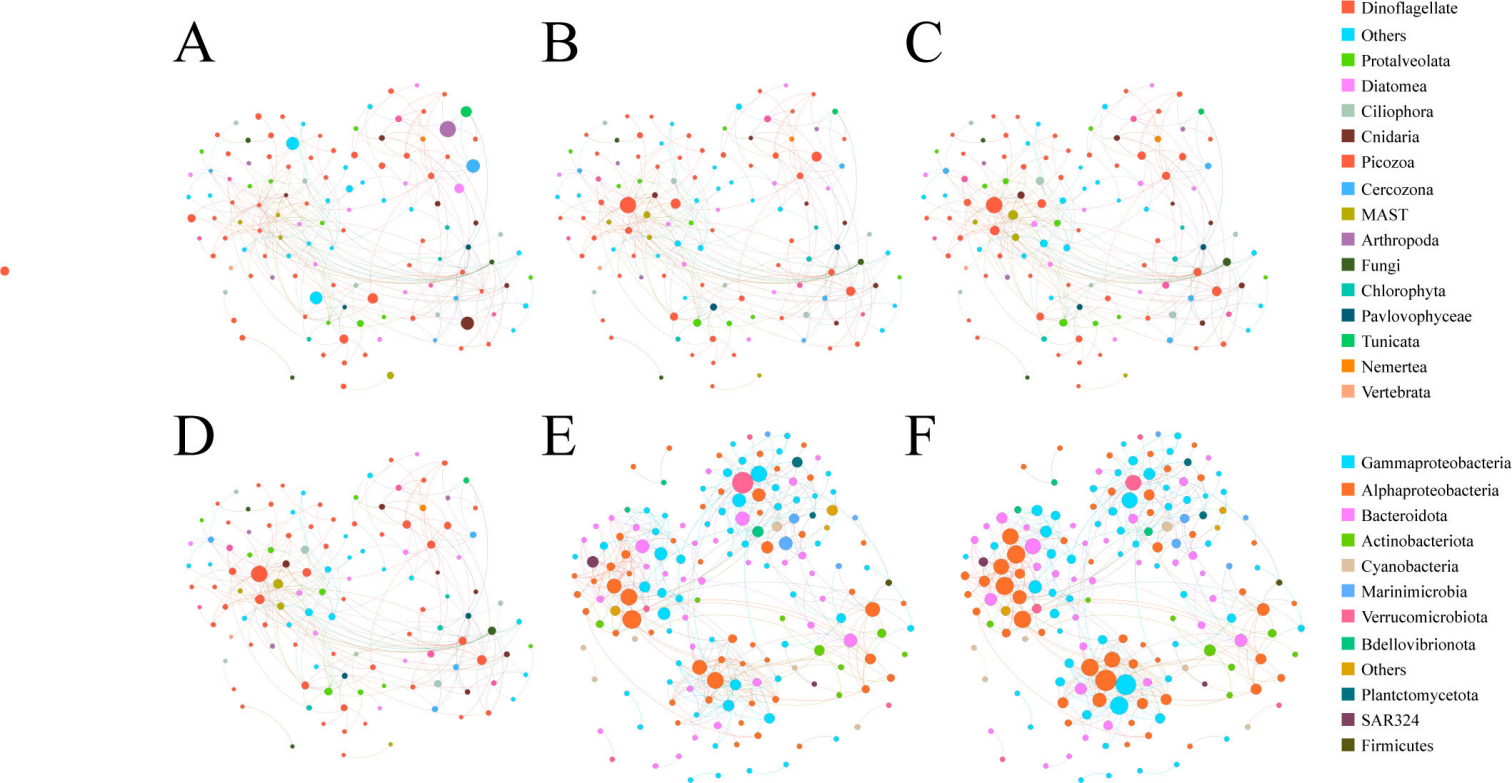
Network modules of eukaryotic plankton communities in terms of (A) abundance, (**B**) betweenness, and (C) degree and prokaryotic plankton communities in terms of (D) abundance, (**E**) betweenness, and (F) degree. In these co-occurrence networks, each node represents an ASV, which corresponds to a microbial taxa. The color of the nodes represents each main category. Edges between nodes are links that indicate an interaction between two nodes, pink are negative links, while green are positive.

A co-occurrence network of prokaryotic and eukaryotic plankton communities was constructed and fitted to a power-law distribution (*P* < 0.001) (Fig. S7), indicating that the community networks were non-random and had ecological significance. The prokaryotic plankton community network was greater than the eukaryotic plankton community in terms of its nodes (173 versus 125), edges (393 versus 241), and modules (14 versus 6) (Fig. 5; Table S3). The prokaryotic plankton networks were more modularized than the microeukaryotic networks (0.650 versus 0.499 for eukaryotic plankton) (Fig. 5). Furthermore, the prokaryotic plankton networks possessed a higher average path distance (4.215 versus 3.789) and average clustering coefficient (0.213 versus 0.058). These results show that any two prokaryotic plankton species in the microbial communities can be linked by a small number of neighboring species, displaying a “small-world” property. There will be few highly connected nodes in small-world systems, allowing for rapid transmission of perturbations (environmental variations) to the whole community. Overall, these results demonstrate that the prokaryotic plankton communities had higher network complexity than microeukaryotes.

Modules of prokaryotic plankton networks were more strongly affected by environmental gradients than microbial prokaryotes (Table S4). Dissolved oxygen, total inorganic nitrogen, nitrite, and silicate were only significantly related to the assemblages of microeukaryotic modules, while latitude, salinity, chl *a*, and DIP were associated with both prokaryotic and eukaryotic plankton modules. Interestingly, latitude, salinity, and chl *a* significantly affected the organization of microbial modules in both positive and negative directions. Diverse taxa, affiliated with many microbial phyla, coexisted at each module and had a variety of node properties. A range of dinoflagellate and diatom species (eukaryotic plankton) were key connectors or “gatekeepers” in contributing to the co-occurrence network structure (Table S5). For example, *Pyrphacus steinii* was not the most abundant microeukaryotes but was associated with high betweenness and degree. Four species of Rhodobacteraceae made a significant contribution to betweenness and degree, whereas *Synechococcus* (Cyanobacteria) dominated the prokaryotic plankton community but had low degree and betweenness.

**Fig 6:**
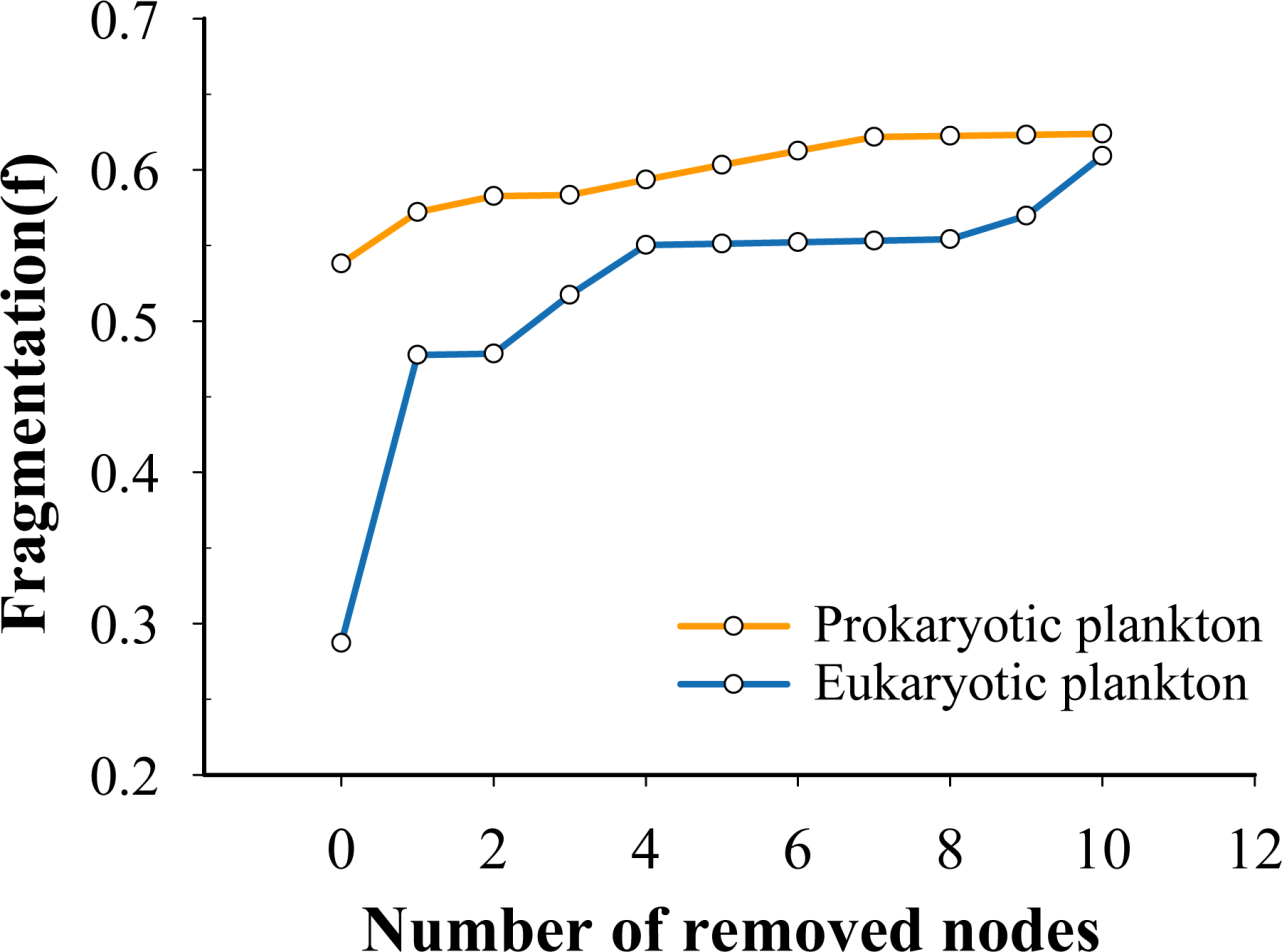
The fragmentation of co-occurrence networks with consecutive removal of 10 nodes with the highest betweenness centrality for eukaryotic and prokaryotic plankton communities.

**Fig 7:**
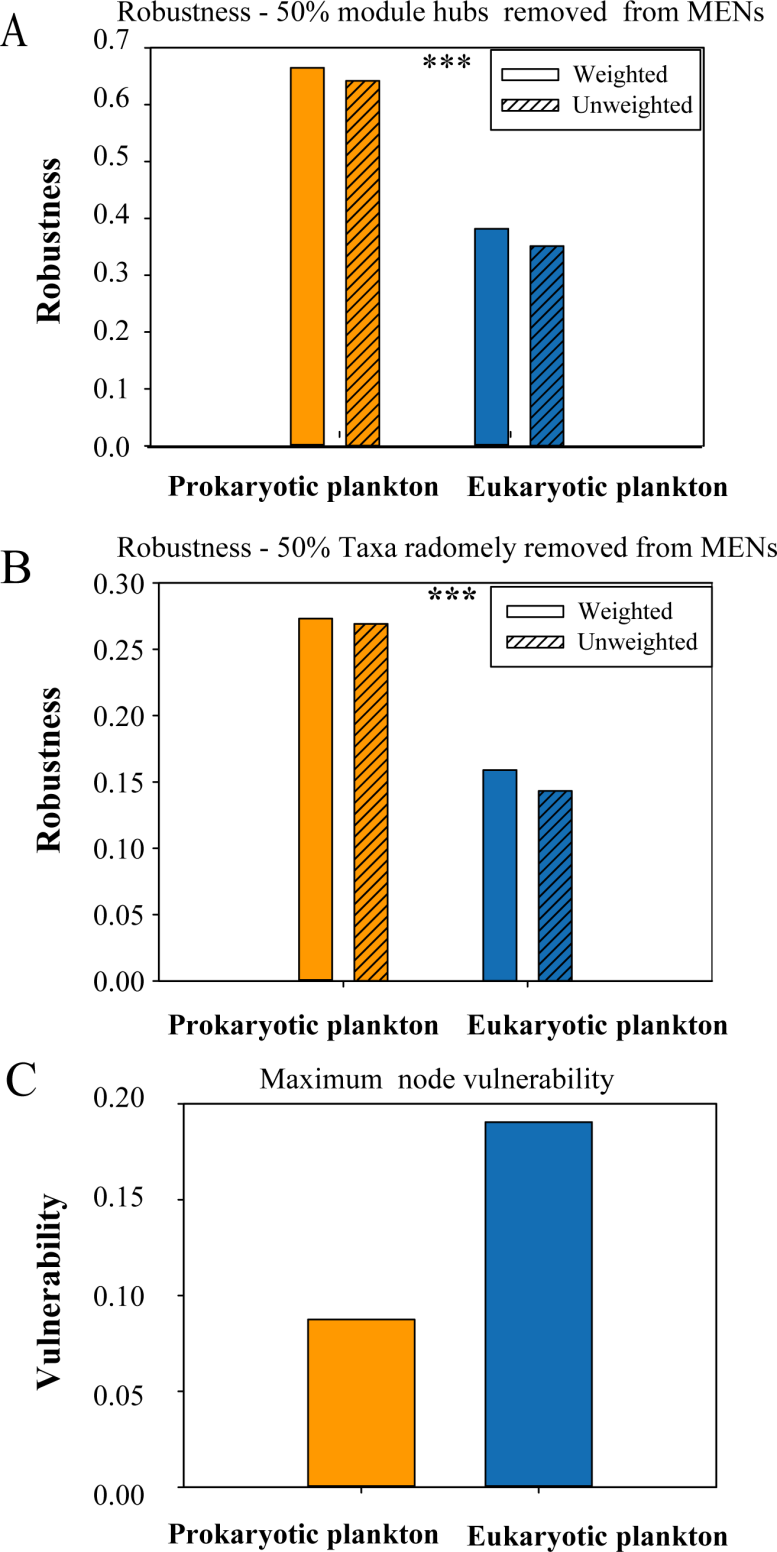
Robustness and vulnerability of microbiomes. (**A**) Robustness measured as the proportion of taxa remained with half module hubs removed from each of the empirical MENs, (**B**) robustness measured as the proportion of taxa remained with 50% of the taxa randomly removed from each of the empirical MENs, (C）network vulnerability measured by maximum node vulnerability in each network. Asterisks denote significance (***P < 0.001).

After removing the top 10 nodes associated with the highest betweenness, microeukaryotes exhibited a higher degree of fragmentation than prokaryotic plankton communities (Fig. 6). Furthermore, the prokaryotic plankton communities displayed high robustness and low vulnerability in comparison to the microeukaryotes (Fig. 7). These results indicate that prokaryotic plankton communities had higher network stability than the microeukaryotes (Fig. 7).

## DISCUSSION

Distinct responses of prokaryotic and eukaryotic plankton communities to the spatial heterogeneity in the Bohai Sea and Yellow Sea were observed in terms of geographical distribution, phylogenetic distance, niche breadth, and community assembly process. (Fig. 1 to 4; Table 1). For example, latitude made a major contribution to shaping the distribution of microeukaryotes and prokaryotic plankton communities (Fig. S4 and S5; Table S2). Results presented here are consistent with prior studies in this region (35), which demonstrated that geographic distance especially latitude could explain the significant heterogeneous distribution of both bacterial and archaeal communities across different taxonomic levels. In addition, microeukaryotic community diversity was more susceptible to DIP and DO gradients compared to the prokaryotic plankton communities (Table 1). The distribution of dinoflagellates was negatively correlated with increased phosphate concentration and latitude (Fig. 2), which means that the lower latitude and oligotrophic conditions (lower phosphate concentration) are beneficial to the survival and bloom of dinoflagellates. Eukaryotic and prokaryotic microbes compete for environmental niches (resources) with differing proliferation strategies in the Bohai Sea and Yellow Sea and thus influence their distribution, survival strategy, and ecological functions (36, 37).

Significant correlations between silicate concentration and latitude with parameters including salinity, dissolved oxygen, nitrogen, and ammonia were observed. These results show that the geographic distribution of microbiomes was not just influenced by the spatial gradients and nutrient availability individually, but rather by their combined influence on the environmental heterogeneity. For example, latitude, salinity, and nutrient availability contribute together to the distribution and associations of microbiome groups. In previous studies, spatial environmental heterogeneity was assumed to increase species diversity by providing more available niche space, resources, and structural complexity, and to provide shelter and refuges from changing environmental conditions, allowing more species to coexist in multiple habitats with improvement to their species persistence (38–40). In addition, the mutual influences between environmental variation and microbial geographic distribution were demonstrated to be complex in this coastal area. Stochastic processes played important roles in the construction of eukaryotic and prokaryotic plankton communities which are dominated by dispersal limitations. Similar results have also been found in the coastal Bohai Sea (41) and a subtropical river-bay system in south China (42), where stochastic patterns significantly affected the microbial communities, although both deterministic and stochastic processes significantly influenced the assembly of microbiomes. Eukaryotic plankton communities were governed more by stochastic processes than the prokaryotic plankton communities, while dispersal limitations determined eukaryotic plankton community structure by stochastic processes (42). Stochastic processes include probabilistic dispersal (random colonization to a new site) and ecological drift (random shift in abundance) (43). In contrast, environmental filtering, where the environment selects against certain species, is considered a major mechanism structuring eukaryotic plankton community in this region, along with other deterministic processes including species competition and trophic interactions (43, 44). Environmental selection is the key driver of the divergence of microbial communities in aquatic environments (35, 45). For example, deterministic factors determine the community composition in the Pearl River estuary (42). Similarly, this study explored significant differences in the distribution and phylogenetic diversity between eukaryotic and prokaryotic plankton communities. Eukaryotic plankton communities in the Bohai Sea and Yellow Sea were mostly influenced by stochastic processes, whereas prokaryotic plankton communities had a significant deviation, suggesting that deterministic processes governed the community assemblage (43, 46).

Microeukaryotic plankton had a greater phylogenetic distance than the prokaryotic plankton, and thus habitat filtering, i.e., local environmental conditions, rather than competition-structured bacterioplankton communities. Moreover, prokaryotic plankton communities exhibited greater environmental niche breadth. Results presented here indicate that habitat generalists made a substantial contribution to determining spatial variation in marine bacterial community composition in areas such as the Baltic Sea (47). Environmental niche breadth generally increased under resource limitation, thus prokaryotic plankton communities are more competitive and have stronger species evolutionary adaptation in response to environmental changes in comparison to eukaryotic plankton (48).

Results presented here show that there are distinct distribution and environmental adaptation strategies for eukaryotic and prokaryotic plankton communities in the Bohai and Yellow Seas. Stochastic processes played an important role in shaping the structure of microbial communities, especially the eukaryotic microorganisms. Deterministic processes, such as nutrient availability, played a leading role in shaping the structure of prokaryotic plankton communities. Prokaryotic plankton communities were associated with a wider environmental breadth and stronger evolutionary environmental adaptation than eukaryotic plankton. Environmental selection and dispersal limitations are basic ecological processes and are incorporated into a better understanding of the mechanisms of microbiome organization in this region (35, 49).

Spatial heterogeneity was observed between abundant and rare subgroups of prokaryotic and eukaryotic plankton communities. Abundant and rare taxa significantly differed in their diversity, distribution, phylogenetic distance, environmental breadth, and community assembly processes. For example, rare subgroups of the prokaryotic plankton community were shaped by geographic distance and ammonia concentration, whereas abundant subgroups were strongly affected by nutrient availability (Table 1). This result is consistent with a study of Chinese inland freshwater ecosystems, indicating that rare bacterial taxa seem to be more limited by local environmental conditions than abundant ones (19). Similar results have also been found in the Mediterranean and Black Seas, where marine plankton microbial assemblages incorporate dynamic, abundant, and rare subcommunities, with contrasting structuring patterns but fairly regular proportions (50). The composition of rare eukaryotic plankton subcommunities was more resource-dependent and more modulated by local environmental selection, as environmental variations are significantly correlated with the dynamics of rare eukaryotic plankton subcommunity rather than the prokaryotic plankton subcommunity.

Environmental variations had no significant impact on the diversity of abundant eukaryotic and prokaryotic plankton subcommunities, although community composition and distribution were significantly influenced. For example, the Shannon-Wiener diversity of abundant prokaryotic microbial communities was strongly affected by chl *a* and salinity gradients, while the diversity of rare subgroups was not influenced by environmental variations. A similar pattern was also found in three subtropical bays of China and Chesapeake Bay, where abundant and rare bacterial taxa differed in their diversity (51, 52).

Stochastic processes contributed greatly to the assembly of microbiome organization, and the relative contribution of ecological process differed across subcommunities. Differing environmental adaptation/organization of abundant and rare bacterioplankton was also observed in Lake Nanhu (53) and the Yangtze River (54). Stochastic processes more strongly shaped the assembly of rare prokaryotic subcommunities than those of rare eukaryotic subcommunities. There were also fewer species dispersal influences on the assembly process of the rare eukaryotic subcommunity than the rare prokaryotic subcommunity. The abiotic factors (such as nutrient availability and temperature) along with biotic factors (such as predation and species competition) are essential in shaping marine microbial communities (42, 51, 53, 55). Our results suggest that rare subcommunities are low in abundance and more likely to be lost or replaced due to ecological drifts in the Bohai Sea and Yellow Sea (4).

Abundant taxa have a greater adaption potential to respond to environmental variation and disturbance along with a broader niche breadth (Fig. 4). This illustrates that abundant taxa are predicted to have strong adaption response to environmental changes during evolutionary processes with increased competitive ability after resources limitation, while rare taxa were in an inferior position for resource competition (48). This also suggests that abundant and rare eukaryotic and prokaryotic plankton communities may have discrepant ecological niches and play different roles in marine ecosystems (31).

In general, deterministic processes and stochastic processes played important roles in shaping the diversity and distribution of rare prokaryotic subcommunities in the Bohai Sea and Yellow Sea. Stochastic processes had leading roles in the distribution of prokaryotic plankton subcommunity, while deterministic processes governed the community assembly of rare eukaryotic plankton subcommunity (Fig. 3H).

Phylogenetic characteristics of abundant and rare eukaryotic and prokaryotic subcommunities may have distinctive sensitivities when facing ongoing environmental changes in the Bohai Sea and Yellow Sea. Abundant microbial subcommunities are being less damaged in response to dynamic environments, and hence have distinct biogeographic distribution compared to the rare subgroups (Fig. 3F and G) (32, 53). Less phylogenetic distance of rare subgroups indicates that specific rare taxa are comparatively weak in preserving ecological niches during environmental perturbations, leading to a distinct biogeographic pattern compared to the abundant subgroups.

Prokaryotic microbiomes, which possess more complex community networks than microeukaryotes, responded differently to the environmental heterogeneity of the Bohai and Yellow Seas. Marine microbiomes expressed differential sensitivity in response to coastal variations (56). For example, microbiomes from warm offshore areas exhibited larger changes in community interactions than nearshore microbes (57). This result is consistent with a study of Chesapeake Bay, which found that estuarine gradients dictate spatiotemporal variations of microbiome networks (35). In addition, prokaryotic plankton are more modularized in their network structure, associated with high connectivity within the community (58). Similarly, prokaryotic plankton exhibits a “small world property,” meaning that any two microbial species in the community can be linked by just a few neighbor species (highly interconnected nodes). This allows perturbations to spread quickly, and potentially alter the performance and properties of the whole network by changing only a small number of connections (22, 59). Compared to prokaryotes, responses of the network module eigengenes to coastal gradients showed strong relationships between microeukaryotic modules and latitude, salinity, and nutrient availability (Table S4). Results presented here illustrate that distinct co-occurrence patterns of species linkages within eukaryotic and prokaryotic plankton communities are present in the Bohai and Yellow Seas under the influence of spatial heterogeneity. Co-occurrence network complexity and stability are crucial to reveal interactions among microbial taxa and stress resistance in coastal areas (60), such as the Chesapeake Bay (43). Microbial community interactions are sensitive to even a slight change in temperature (61). Thus, environmental disturbances could influence the survival and adaption ability of microbiomes and thereby the threat to ecosystem functions.

The stability and resilience of eukaryotic and prokaryotic microbial communities differed in the Bohai and Yellow Seas. Generally, it is commonly believed that network complexity begets stability, this study supports this theory by demonstrating a strong positive correlation between network stability and network complexity (27). Molecular ecological networks of prokaryotic plankton are significantly more robust than those of eukaryotic plankton for both the simulations of random species removal and targeted removal. In general, this study shows that prokaryotic microbial communities have higher molecular network complexity and stability compared to the microeukaryotes and this indicates a stronger anti-interference ability. Prokaryotic microbial communities tend to be more stable than microeukaryotes under environmental disturbance or pollution due to anthropogenic influences, especially when this results in the loss of some of its key species with a high degree/betweenness.

The hub species and gatekeepers of the community networks contribute to the association of prokaryotic/eukaryotic microbiomes. The absence of key species may cause the decomposition of modules and networks, which play critical roles in maintaining the stability of microbial community interactions (62). For example, several ASVs affiliated with Rhodobacteraceae, SAR116 clade, and Dinoflagellata taxa (*Pyrphacus* sp. and *Fragilidium* sp.), which had low relative abundances but a high degree and betweenness, played critical roles in holding the network together. Based on the stepwise responsiveness of the fragmentation analyses to remove species with the highest betweenness, microeukaryotes had higher levels of fragmentation in comparison with prokaryotic microbiomes. Prokaryotic communities also had higher stability and stronger resilience than eukaryotic plankton communities (23, 63, 64). These results highlight that removing “gatekeeper” species such as *Pelagodinium* sp. from microeukaryote communities may lead to network fragmentation (63). These “gatekeeper species” have a comparatively low abundance, but are critical in regulating the ecological functions of microbiomes in the Bohai and Yellow Seas (63, 65).

In summary, architectural patterns and mechanisms underlying the network complexity and stability of prokaryotic and eukaryotic plankton communities were systematically explored in the Bohai Sea and Yellow Sea. Environmental heterogeneity structured microbial communities into a clear spatial pattern and provided a better explanation for the variability of prokaryotic plankton, with complex direct/indirect relationships, in comparison to the eukaryotic plankton. Environmental gradients did not alter the diversity of abundant subcommunities, but salinity gradients had constructing impacts on the richness of rare prokaryotic microbial subgroups and the rare microeukaryotic subgroups. Interestingly, the spatial gradients and nutrient availability strongly shaped the distribution of abundant subgroups compared to the rare subgroups. These results show the distinct responses and survival strategies of prokaryotic and eukaryotic plankton communities and the abundant and rare subgroups. Stochastic processes made a major contribution to microbiome dynamics but deterministic processes governed the prokaryotic plankton community organization with a lower phylogenetic turnover rate. Rare subgroups had a noticeably higher phylogenetic distance and lower niche breadth than the corresponding abundant subgroups. Prokaryotic plankton communities exhibited greater niche breadth than eukaryotic plankton communities. These results can be further explained by the long-term ecological evolution of microbial communities in the Bohai Sea and Yellow Sea. Ecological niches of both eukaryotic and prokaryotic plankton were replenished during the adaptation process with distinct patterns. Prokaryotic microbiomes exhibited stronger network modularity and greater community complexity and stability than eukaryotic microbiomes Network stability characteristics demonstrated greater resilience in prokaryotic plankton communities but microeukaryotic communities were more susceptible to environmental perturbations. In general, this provides important insight on the construction of ecological models to simulate the effects of environments or climate change on the characteristics and homeostasis of coastal microbiomes, and supporting the preservation of sustainability and ecological functions of coastal oceans.

## MATERIALS AND METHODS

### Sampling collection

To compare eukaryotic and prokaryotic plankton communities of the Bohai Sea and Yellow Sea, a total of 38 surface water samples were collected from 26 June to 1 August 2019 (32°−39.5°N, 119.9°−124.5687°E) (Fig. S1). Water samples were collected with 12 Go-Flo bottles equipped with a SeaBird CTD (SBE 9/11 plus and SBE 25 plus). At the same time, CTD data including temperature (T), salinity, pressure, DO, nitrates, and chlorophyll *a* were recorded on board. Water samples were refrigerated at −20℃ for determination of nutrient concentration (NO_3_^−^–N, NO_2_^−^–N, PO_4_^3−^–P, NH_4_^+^, and SiO_3_^2−^–Si) with a Bran + Luebbe Auto Analyzer 3 using colorimetric methods. At each sampling site, 8 L of seawater was collected from the surface and poured into a prewashed plastic bucket. Four liters of the water for phytoplankton analysis was passed through 3 µm and 0.22 µm membranes, respectively, with a vacuum pump (low pressure, <100 mm Hg), each sample had two replicates. The 3 µm and 0.22 µm membranes were stored at −80°C for further analyses.

### DNA extraction and sequencing

Total DNA was extracted by the lysozyme-SDS-phenol/chloroform method and amplified using barcoded primers targeting the 16S and 18S ribosomal RNA (rRNA) genes. High-throughput sequencing of 16S and 18S rRNA genes was performed at Magigene (Magigene Biotechnology, Guangzhou, China) on an Illumina Nova6000 platform (paired-end 250 bp mode), following the manufacturer’s guidelines. Genomic DNA was amplified by PCR with the primer set 515F (5′-GTGYCAGCMGCCGCGGTAA-3′) and 806R (5’- GGACTACNVGGGTWTCTAAT-3′) (66, 67). The V3–V4 region of the 18 rRNA gene was amplified using the primers set 528F (5′-GCGGTAATTCCAGCTCCAA-3′) and 706R (5’- AATCCRAGAATTTCACCTCT-3′) (68). After obtaining the offline data, the company preprocesses the sequencing data. Raw reads were quality filtered and taxonomically assigned through the microbiome bioinformatics platform (QIIME 2 pipeline, https://docs.qiime2.org/2021.4/interfaces/q2cli/). Sequence processing was performed using the quantitative insights into the pipeline Qiime 2 (2021.4). After cutting off the sequences of barcodes and primers, features were filtered to remove unqualified data (ASV occurred in less than 2 samples or frequency smaller than 5). Each representative ASV was aligned against the SILVA 138-99 reference alignment using the QIIME2 taxonomy classifier for taxonomic affiliations. Unassigned sequences were removed for microeukaryotes plankton. Unassigned samples and reads affiliated with chloroplast were removed for prokaryotic plankton. A phylogenetic tree was constructed with FastTree by QIIME2. A randomly selected subset of 44,800 sequences from the eukaryotic plankton community (64,133 for the prokaryotic plankton community) from each sample was used to normalize the sequencing effort across samples. The final total data set retained 1,945 ASVs from eukaryotic plankton and 1,818 ASVs from the prokaryotic plankton community. The rarefaction curve showed sufficient sequencing depth for both eukaryotic and prokaryotic plankton communities (Fig. S1).

### Definition of abundant and rare subcommunities

Classifications of abundant and rare microbiomes were set according to previous studies on abundant and rare eukaryotic plankton communities (4, 54, 69). Abundant and rare taxa were classified depending on the cutoff of relative abundance and occurrence of samples, abundant species had relative abundances greater than 1% in at least 10% of all samples, or a summed abundance greater than 20% in all stations (4), 47 ASVs from the eukaryotic plankton community and 50 ASVs from the prokaryotic plankton community were identified as abundant taxa. Rare species were classified as a summed relative abundance of less than 0.5% across all samples; there were 1,444 ASVs for eukaryotic plankton and 1,297 ASVs for prokaryotic plankton. The remaining species were considered as moderate taxa.

### Multiple regression model

An MRT analysis attempts to predict dependent variables; these are based on the value of two or more independent variables in complex ecological data (57). In this study, MRT used environmental gradients as a classification to describe and predict the relationship between two explanatory variable matrices using the “mvpart” package of RStudio 4.1.1. Results given by MRT can be further classified as environmental parameters in groups.

### Distribution of microbiomes and its relation with environmental factors

Spatial effects on alpha-diversity (Shannon-Wiener index, richness, Pielous’s evenness, and Faith’s phylogenetic diversity) were examined with Spearman’s correlation statistical analysis. One-way analysis of variance (ANOVA) and Turkey HSD tests were carried out to determine the dissimilarity between environmental variations and microbial diversity. Non-metric multidimensional scaling (NMDS) was performed using Bray-Curtis distance matrices to describe variations in both eukaryotic and prokaryotic plankton and their subcommunity over geospatial scales (70). Mantel tests give comparisons between two distance matrices using Euclidean methods, pairwise geographic distances can be generated from environmental vectors (longitude and latitude) of each site. The ANOVA, Mantel, and NMDS analyses were performed using “aov,” “mantel,” and “metaMDs” functions in the vegan R package (R version 4.1.1).

### Redundancy analysis and piecewise SEM statistical analysis

RDA was carried out in R studio (R version 4.1.1) with the vegan package to analyze the relationships between environmental parameters and the distribution of eukaryotic and prokaryotic plankton communities. This analysis was calculated based on Hellinger-transformed ASV data and an environmental parameter data set. Explanatory variables with strong linear dependencies (variance inflation factors > 10) were removed first (71). To identify the “best” explanatory variables and decide when to stop the selection, step forward selection with the function “ordistep” in the vegan R package was applied. The taxa with a goodness-of-fit of at least 0.49 in the ordination plane formed by axes 1 and 2 are shown in the RDA plot (71). The significance of the RDA model was further tested by ANOVA, based on 999 permutations. This analysis included the major environmental parameters that shape eukaryotic and prokaryotic plankton community composition in the Bohai and Yellow Seas.

A piecewise SEM was applied to further analyze the direct and indirect influences of spatial heterogeneity on the composition and distribution of microbial communities (72). Direct and indirect partition effects on interrelated environmental variables were estimated and the relative strength between them was calculated. In the piecewise SEM model, a priori model was built based on the known effects and correlations among environmental parameters and dynamics of microbial communities. Then, a maximum likelihood goodness-of-fit test was used in model fitting with a non-significant *P* value indicating a well-fit model. Thus, both the direct and indirect effects of each environmental parameter relative to other variables can be observed and the strength of multiple effects can be determined.

### Neutral community model

To clarify the potential importance of stochastic processes on the assembly of eukaryotic and prokaryotic plankton communities, a neutral community model was applied based on the occurrence frequency of prokaryotic and eukaryotic microbial ASVs and their rare subgroups, respectively (73). The neutral model, that describes the stochastic balance between the immigration, speciation, emigration, and extinction of organisms, was carried out in R studio (R version 4.1.1) using “Hmisc,” “minpack.lm,” and “stats4” packages.

### Phylogenetic clustering and niche breadth analysis

To estimate the phylogenetic clustering of eukaryotic and prokaryotic microbial communities and their subgroups, a framework was used to untangle the importance of ecological processes (35, 74). SES MNTD was calculated to reflect the phylogenetic clustering which reflects whether species cluster closer to the tips of the phylogeny (75, 76), βMNTD, as evaluated pairwise phylogenetic turnover between microbial communities, can be defined as the observed mean phylogenetic distance to the closest relative between different microbial communities (74, 76). β nearest taxon distance (βNTI) values were applied to calculate the relative importance of ecological drift and to measure variations in phylogenetic and taxonomic diversity (43, 76). βNTI, which is the standardized effect size of βMNTD, was calculated as the difference between observed βMNTD and the mean of the null distribution of βMNTD normalized by its standard deviation (74). SES MNTD, βMNTD, and βNTI were calculated using the “ses. mntd” and “comdistnt” functions from “picante” R packages, showing phylogenetic clustering, phylogenetic distance, and phylogenetic turnover rate, respectively (77). βNTI with significant deviation (|β NTI| > 2) indicates that a deterministic process is dominant and a non-significant deviation (|β NTI| < 2) indicates that a stochastic process is dominant, β NTI < −2 is referred to as a homogeneous selection while β NTI > 2 is referred to as a variable selection (43, 74, 78).

Environmental niche breadth statistical analysis was also carried out in RStudio (R version 4.1.1) using the “niche.width” function from the “spaa” package. This analysis can help determine the threshold of niche breadth of eukaryotic and prokaryotic plankton communities and their subgroups in response to environmental variations and gives a visualization of their habitat specialization across biographic gradients (79, 80).

### Co-occurrence network construction and topology properties

The networks for both eukaryotic and prokaryotic plankton communities were constructed based on the Molecular Ecological Network Analysis Pipeline (MENAP) (22, 81–83). The algorithms employed by the MENAP platform have the advantage of reliably removing noise from fly-random, system-specific features. In this process, node-level topological properties, including degree and betweenness, were characterized, while modular analysis were done within processes of the MENAP platform. The nodes with a high degree of betweenness are important for network structure and persistence because they hold the network together (63). Topologies, such as the number of nodes and links, average clustering coefficient, average path distance (*L*), average clustering coefficient, and modularity are important indicators of network complexity (48). Average path distance (*L*) is the average shortest path between all pairs of species. The average clustering coefficient (avgCC) is the average fraction of pairs of species one link away from a species that are also linked to one another. For network visualization, 125 and 173 ASVs for eukaryotic and prokaryotic plankton communities were obtained, respectively. Gephi (version 0.9.2) has been applied for topological properties and network fragmentation analysis. Finally, robustness and vulnerability, key factors of network stability analysis, and important predictors of eukaryotic and prokaryotic community stability were estimated using R studio (R version 4.1.1) (22).

## Data Availability

Raw sequence data are available in the NCBI database under the accession numbers PRJNA946683 (prokaryotic plankton) and PRJNA945593 (eukaryotic plankton).

## SUPPLEMENTAL MATERIAL

Supporting information msphere.00395-24-s0001.docx Figures S1-S7 and captions for Tables S1-S5.

Supplemental tables msphere.00395-24-s0002.docx Tables S1-S5.

ASM does not own the copyrights to Supplemental Material that may be linked to, or accessed through, an article. The authors have granted ASM a non-exclusive, world-wide license to publish the Supplemental Material files. Please contact the corresponding author directly for reuse.
