# Supplementary material for: Community organization and network complexity and stability: contrasting strategies of prokaryotic versus eukaryotic microbiomes in the Bohai Sea and Yellow Sea
Source: mSphere. 2024 Aug 13;9(9):e00395-24. doi: 10.1128/msphere.00395-24 (PMC11423591; doi:10.1128/msphere.00395-24)
Supplement: Supporting information — Figures S1-S7 and captions for Tables S1-S5. [file msphere.00395-24-s0001.docx]

Community organization and network complexity and stability: Contrasting strategies of prokaryotic versus eukaryotic microbiomes in the Bohai Sea and Yellow Sea

**Xiaoxiao Wang^a,b^, Hualong Wang^a,b,*^, Yantao Liang^a,b^, Andrew Mcminn ^a,c^, Min Wang ^a,b,*^**

^a^ College of Marine Life Sciences, Institute of Evolution and Marine Biodiversity, Frontiers Science Center for Deep Ocean Multispheres and Earth System, Key Lab of Polar Oceanography and Global Ocean Change, Ocean University of China, Qingdao 266003, China

^b^ UMT-OUC Joint Center for Marine Studies, Qingdao 266003, China

^c^ Institute for Marine and Antarctic Studies, University of Tasmania, Hobart, 7000, Australia

* Corresponding author: mingwang@ouc.edu.cn (Min Wang), wanghualong@ouc.edu.cn (Hualong Wang)


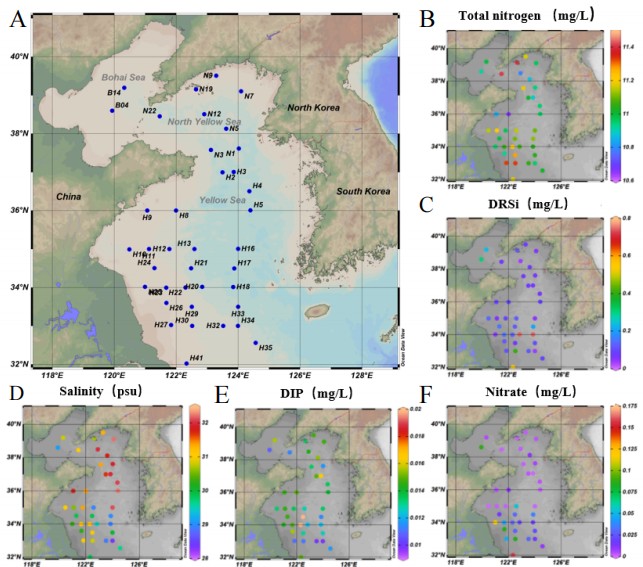


**Figure S1** A) The map of sampling sites and environmental layers in the Bohai Sea and Yellow Sea. Environmental layers obtained from Ocean Data View and indicate concentrations of B) Total nitrogen (mg/L), C) DRSi (mg/L), D) Salinity (psu), E) DIP (mg/L) and F) Nitrate (mg/L). Color gradations show relative values in each layer.


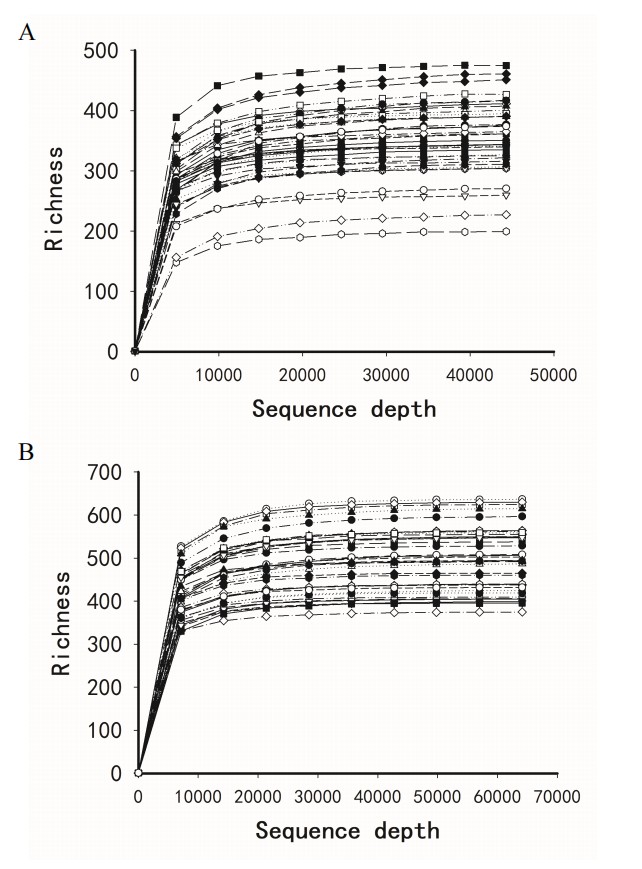


**Figure S2** Alpha rarefaction curve of A) eukaryotic plankton at sampling depth of 44600 B) prokaryotic plankton at sampling depth of 64133.

**
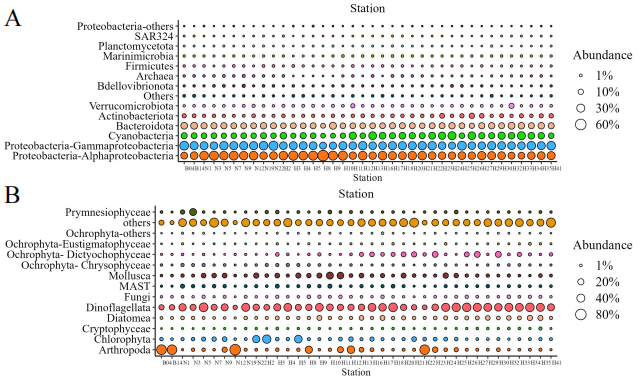
**

**Figure S3** Spatial distribution and relative abundance of A) dominant prokaryotic plankton communities across all stations B) dominant eukaryotic plankton communities across all stations. Bubble size represents relative abundances of total sequence reads for each sample.


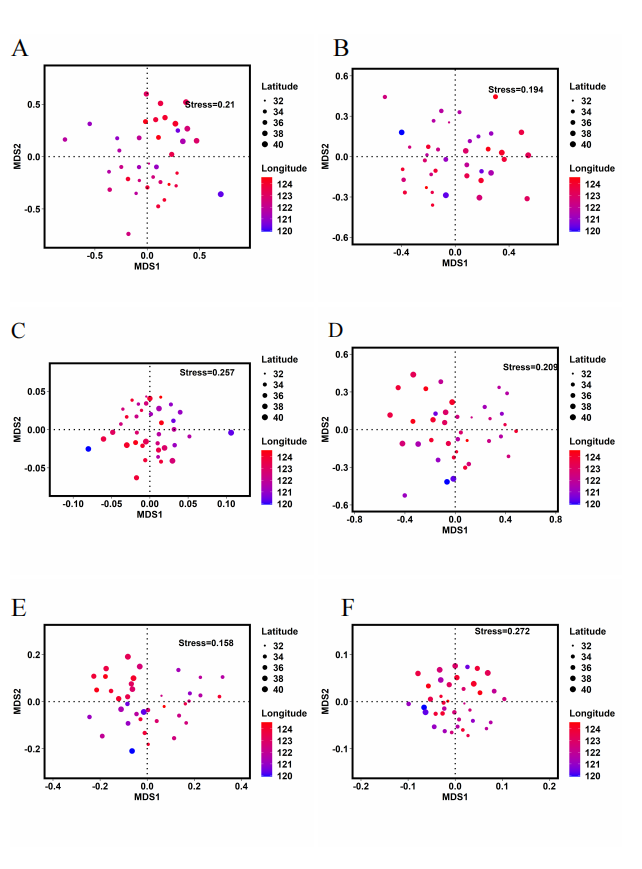
 **Figure S4** Nonmetric multidimensional scaling (NMDS) plot indicating influence of geographic pattern on abundance of eukaryotic and prokaryotic plankton communities and their subgroups in the Bohai Sea and Yellow Sea. NMDS plots are listed as A) eukaryotic plankton community, B) abundant eukaryotic subgroup, C) rare eukaryotic subgroup, D) prokaryotic plankton community, E) abundant prokaryotic subgroup, F) rare prokaryotic subgroup. Sampling stations are color-coded (representing longitude gradient) and symbol shapes represent latitude gradient.

**
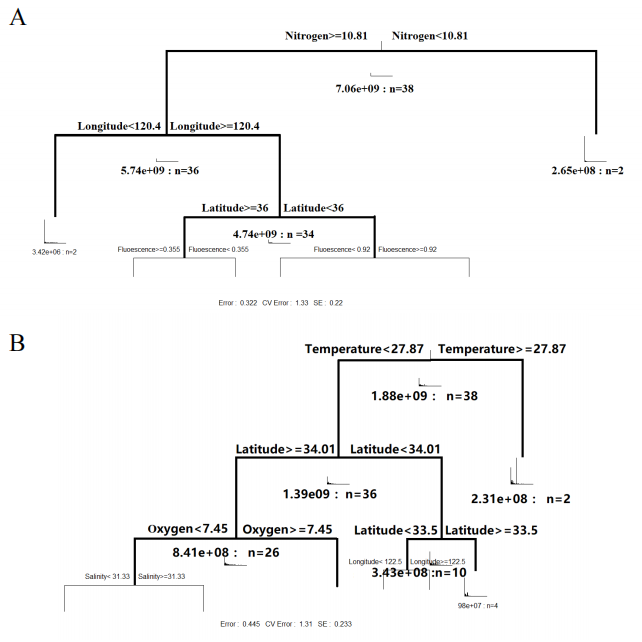
 Figure S5** Multiple regression tree model of A) eukaryotic plankton community and B）prokaryotic plankton community based on multiple independent variables (environmental factors).

**
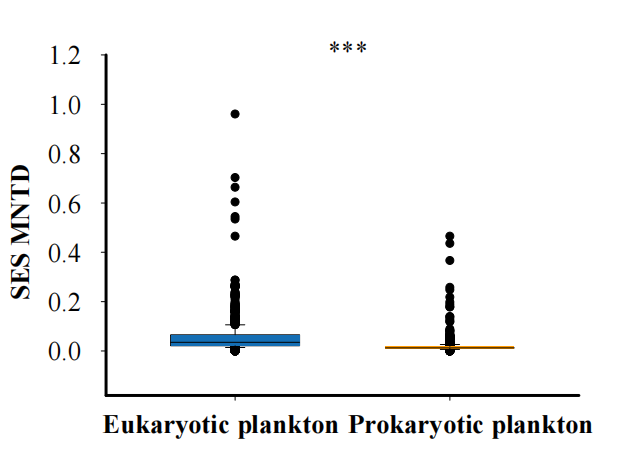
 Figure S6** Phylogenetic evenness based on SES. MNTD matrix between eukaryotic and prokaryotic microbial communities.

**
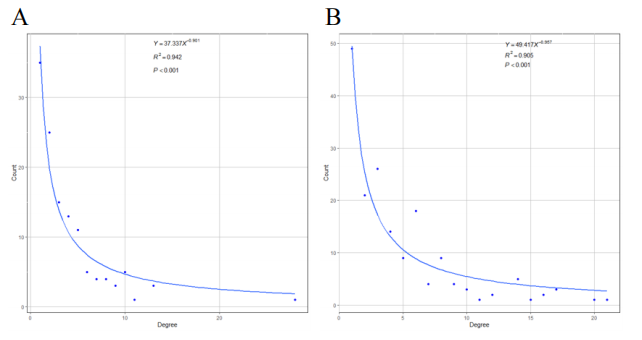
**

**Figure S7** Power law distribution of A）eukaryotic plankton communities and B）prokaryotic plankton communities.

**Table S1** Spearman' correlation statistical analysis showing the effects of temperature, salinity and other environmental factors on the alpha diversity of eukaryotic and prokaryotic plankton communities and their subgroups.

**Table S2** ANOSIM test of community composition of eukaryotic and prokaryotic plankton communities between subgroups or among subgroups of key environmental factors.

**Table S3** Topological properties of prokaryotic and eukaryotic plankton co-occurrence networks in the Bohai Sea and Yellow Sea.

**Table S4** Visualization of eigengene network representing module trait relationships among the modules MEs and the environmental variables for eukaryotic and prokaryotic plankton communities.

**Table S5** Key species associated with relatively high degree, betweenness or abundance in plankton co-occurrence network.
