# Supplementary material for: Community organization and network complexity and stability: contrasting strategies of prokaryotic versus eukaryotic microbiomes in the Bohai Sea and Yellow Sea
Source: mSphere. 2024 Aug 13;9(9):e00395-24. doi: 10.1128/msphere.00395-24 (PMC11423591; doi:10.1128/msphere.00395-24)
Supplement: Supplemental tables — Tables S1-S5. [file msphere.00395-24-s0002.docx]

| **Table S1. Spearman' correlation statistical analysis showing the effects of temperature, salinity and other environmental factors on the alpha diversity of eukaryotic and prokaryotic plankton communities and their subgroups. Note: Asterisks denote levels of significance (**p* < 0.05; ***p* <0 .01; ****p* <0 .001).** | | | | | | | | | | | | | | | | | | | | |
| --- | --- | --- | --- | --- | --- | --- | --- | --- | --- | --- | --- | --- | --- | --- | --- | --- | --- | --- | --- | --- |
| **Environmental variables** | **Eukaryotic plankton** | | | | | | | | | | **Prokaryotic plankton** | | | | | | | | | |
|  | **All** | | | | **Abundant** | | | **Rare** | | | **All** | | | | **Abundant** | | | **Rare** | | |
|  | Pielou's evenness | Richness | Shannon-Wiener | Faith_pd | Pielou's evenness | Richness | Shannon-Wiener | Pielou's evenness | Richness | Shannon-Wiener | Pielou's evenness | Richness | Shannon-Wiener | Faith_pd | Pielou's evenness | Richness | Shannon-Wiener | Pielou's evenness | Richness | Shannon-Wiener |
| Longitude | 0.1338 | -0.0737 | 0.0752 | -0.118 | 0.1982 | 0.1731 | 0.1874 | 0.1069 | -0.0459 | 0.0428 | 0.202 | -0.028 | 0.103 | -0.007 | 0.179 | 0.083 | 0.209 | 0.001 | -0.117 | -0.129 |
| Latitude | -0.1461 | -0.3408* | -0.2386 | -0.208 | -0.2535 | -0.3038 | -0.2417 | 0.6030*** | 0.3066 | 0.3993* | 0.209 | -0.077 | 0.12 | -0.01 | 0.083 | -0.013 | 0.073 | -0.152 | -0.068 | -0.157 |
| Salinity | 0.753 | 0.0732 | 0.034 | -0.207 | -0.0382 | 0.1832 | -0.0062 | 0.2568 | 0.3246* | 0.3183 | -0.026 | -0.289 | -0.163 | -0.214 | -0.139 | 0.131 | -0.12 | -0.249 | -0.3338* | -0.4284** |
| Temperature | 0.2012 | -0.1229 | 0.1511 | -0.0143 | 0.2494 | 0.0199 | 0.2594 | -0.096 | 0.0585 | 0.024 | -0.174 | -0.018 | -0.105 | -0.085 | -0.166 | 0.208 | -0.134 | -0.061 | -0.037 | -0.079 |
| DO | -0.0874 | -0.21 | -0.1312 | -0.125 | -0.1406 | -0.3932* | -0.1715 | 0.3715* | 0.0091 | 0.1019 | 0.007 | 0.005 | 0.008 | 0.017 | -0.04 | -0.277 | -0.07 | -0.106 | 0.07 | 0.061 |
| Chl *a* | -0.0627 | 0.3052 | 0.0406 | 0.259 | -0.002 | -0.0008 | -0.0154 | -0.0892 | -0.1715 | -0.1667 | -0.215 | 0.296 | -0.002 | 0.271 | -0.101 | -0.128 | -0.115 | 0.028 | 0.3954* | 0.4018* |
| Total nitrogen | -0.1294 | 0.0467 | -0.0844 | 0.143 | -0.1224 | -0.0898 | -0.1505 | -0.0846 | -0.2266 | -0.2014 | 0.191 | 0.169 | 0.19 | 0.197 | 0.248 | -0.246 | 0.208 | 0.194 | 0.205 | 0.293 |
| Inorganic N | -0.0297 | 0.305 | 0.0548 | 0.274 | 0.0379 | 0.1169 | 0.0368 | -0.4330** | -0.1673 | -0.2592 | -0.272 | 0.073 | -0.139 | 0.035 | -0.195 | 0.022 | -0.185 | 0.062 | 0.123 | 0.161 |
| Nitrite | 0.2125 | 0.3965* | 0.2971 | 0.3410* | 0.273 | 0.2083 | 0.2565 | -0.4424* | -0.409 | -0.5109** | -0.226 | -0.045 | -0.186 | 0.06 | -0.269 | -0.202 | -0.281 | -0.14 | 0.091 | 0.075 |
| Nitrate | -0.0113 | 0.1572 | 0.0476 | -0.166 | 0.07 | 0.0351 | 0.0668 | -0.2789 | -0.1773 | -0.1773 | -0.202 | 0.084 | -0.092 | -0.003 | -0.146 | -0.042 | -0.135 | 0.044 | 0.128 | 0.168 |
| Ammonia | 0.075 | 0.1703 | 0.1277 | 0.154 | 0.1361 | 0.1162 | 0.1178 | -0.5418*** | -0.1476 | -0.2704 | -0.28 | 0.011 | -0.179 | 0.006 | -0.13 | 0.174 | -0.108 | 0.136 | -0.014 | 0.072 |
| DIP | -0.298 | -0.2523 | -0.0776 | -0.192 | -0.152 | -0.0677 | -0.1474 | 0.0626 | 0.1227 | 0.0513 | -0.164 | -0.213 | -0.219 | -0.205 | -0.213 | 0.019 | -0.212 | -0.22 | -0.221 | -0.298 |
| DRSi | -0.14 | 0.107 | -0.1141 | 0.126 | -0.0924 | 0.046 | -0.104 | -0.3304 | -0.2216 | -0.2884 | 0.043 | 0.096 | 0.075 | 0.084 | 0.069 | -0.041 | 0.053 | 0.278 | 0.16 | 0.25 |

| **Table S2. ANOSIM test of community composition of eukaryotic and prokaryotic plankton communities between subgroups or among subgroups of key environmental factors. Note: Asterisks denote levels of significance (**p* < 0.05; ***p* <0 .01; ****p* <0 .001).** | | | | | | | | |
| --- | --- | --- | --- | --- | --- | --- | --- | --- |
| **Environmental variables** | **Classification** | | **Prokaryotic plankton** | | | **Eukaryotic plankton** | | |
| **Longitude** | Between Groups | | F | | | F | | |
|  |  | Subgroups | Middle | East |  | Middle | East |  |
|  | 119.9297 - 122.5 | West | 0.0394 | 0.0364 |  | 0.0734 | 0.0150* |  |
|  | 122.5 - 123.7 | Middle |  | 0.0077 |  |  | -0.0365 |  |
|  | 123.7 - 124.5687 | East |  |  |  |  |  |  |
|  | Among groups | | 0.02853 | | | 0.07002 | | |
| **Latitude** |  | Subgroups | Middle | Medium High | High | Middle | Medium High | High |
|  | 32.0215 - 34.01 | Low | 0.2908** | 0.7019*** | 0.6759** | 0.2254** | 0.4183*** | 0.7317** |
|  | 34. 01 - 36 | Middle |  | 0.1774** | 0.1111 |  | 0.2194** | 0.4780** |
|  | 36 - 38.8 | Medium High |  |  | 0.1557 |  |  | 0.0045 |
|  | 38.8 - 39.5705 | High |  |  |  |  |  |  |
|  | Among groups | | 0.3682*** | | | 0.314*** | | |
| **Temperature** |  | Subgroups | Moderate | High |  | Moderate | High |  |
|  | 24.138 - 26.34 | Low | -0.02639 | 0.5139 |  | -0.00373 | 0.0201 |  |
|  | 26.34 - 27.87 | Moderate |  | 0.4037 |  |  | -0.1424 |  |
|  | 27.87 - 28.472 | High |  |  |  |  |  |  |
|  | Among groups | | 0.05936 | | | -0.0174 | | |
| **Chl a** |  | Subgroups | Medium | High |  | Medium | High |  |
|  | 0.363 - 0.92 | Low | 0.1355* | 0.3396** |  | 0.269*** | 0.2589* |  |
|  | 0.92 - 1.818 | Medium |  | 0.2259* |  |  | 0.0083 |  |
|  | 1.818 - 4.878 | High |  |  |  |  |  |  |
|  | Among groups | | 0.07821* | | | 0.2032** | | |
| **Total nitrogen** |  | Subgroups | High |  |  | High |  |  |
|  | 10.659 - 11.03 | Low | 0.0782* |  |  | 0.07718* |  |  |
|  | 11.03 - 11.41 | High |  |  |  |  |  |  |
|  | Among groups | | 0.1912** | | | 0.0772* | | |

| **Table S3. Topological properties of prokaryotic and eukaryotic plankton co-occurrence networks in the Bohai Sea and Yellow Sea.** | | |
| --- | --- | --- |
| **Network indexes** | **Eukaryotic plankton** | **Prokaryotic plankton** |
| Average clustering coefficient (avgCC) | 0.058 | 0.213 |
| Average path distance (GD) | 3.789 | 4.215 |
| Geodesic efficiency (E) | 0.315 | 0.288 |
| Harmonic geodesic distance (CD) | 3.173 | 3.468 |
| Density (D) | 0.031 | 0.026 |
| Efficency | 0.974 | 0.973 |
| Modularity(fast_greedy) | 0.499 | 0.65 |

| **Table S4. Visualization of eigengene network representing module trait relationships among the modules MEs and the environmental variables for eukaryotic and prokaryotic plankton communities. Note: Asterisks denote levels of significance (**p* < 0.05; ***p* <0 .01; ****p* <0 .001).** | | | | | | | | | | | | | | |
| --- | --- | --- | --- | --- | --- | --- | --- | --- | --- | --- | --- | --- | --- | --- |
|  |  | **Latitude** | **Longitude** | **Temperature** | **Salinity** | **Dissolved Oxygen** | **Chl *a*** | **Total Nitrogen** | **Total Inorganic Nitrogen** | **Nitrite** | **Nitrate** | **Ammonia** | **DIP** | **Silicate** |
| **Eukaryotic plankton** | **Module 1** | 0.44 | 0.32 | -0.074 | 0.54 | 0.17 | -0.33 | -0.17 | -0.32 | -0.19 | -0.26 | -0.27 | 0.1 | -0.31 |
|  |  | ** |  |  | *** |  |  |  |  |  |  |  |  |  |
|  | **Module 2** | -0.56 | -0.3 | 0.063 | -0.15 | -0.4 | 0.49 | 0.0028 | 0.56 | 0.59 | 0.49 | 0.23 | 0.019 | 0.054 |
|  |  | *** |  |  |  | * | ** |  | *** | *** |  |  |  |  |
|  | **Module 3** | -0.54 | 0.35 | 0.2 | -0.62 | 0.042 | 0.32 | 0.071 | 0.085 | 0.0076 | -0.0016 | 0.28 | -0.57 | 0.12 |
|  |  | *** | * |  | *** |  |  |  |  |  |  |  | *** |  |
|  | **Module 4** | -0.51 | 0.37 | 0.15 | -0.64 | -0.11 | 0.32 | 0.12 | 0.2 | -0.072 | 0.072 | 0.49 | -0.4 | 0.45 |
|  |  | ** | * |  | *** |  |  |  |  |  |  | ** | * | ** |
| **Prokaryotic plankton** | **Module 1** | -0.13 | -0.053 | 0.021 | -0.24 | 0.049 | 0.041 | 0.081 | 0.031 | -0.23 | -0.047 | 0.32 | 0.022 | 0.23 |
|  | **Module 2** | -0.4 | -0.21 | -0.17 | -0.23 | -0.29 | 0.14 | 0.28 | 0.17 | 0.18 | 0.085 | 0.26 | 0.18 | 0.11 |
|  |  | * |  |  |  |  |  |  |  |  |  |  |  |  |
|  | **Module 3** | 0.43 | 0.097 | -0.022 | 0.44 | -0.015 | -0.43 | -0.17 | -0.19 | -0.055 | -0.12 | -0.25 | 0.086 | -0.093 |
|  |  | ** |  |  | ** |  | ** |  |  |  |  |  |  |  |
|  | **Module 4** | 0.14 | 0.33 | 0.25 | -0.16 | 0.03 | -0.12 | -0.18 | -0.17 | -0.26 | -0.1 | -0.22 | -0.33 | -0.2 |
|  |  |  |  |  |  |  |  |  |  |  |  |  | * |  |

| **Table S5. Key species associated with relatively high degree, betweenness or abundance in microbial co-occurrence network.** | | | | | | | | | | |
| --- | --- | --- | --- | --- | --- | --- | --- | --- | --- | --- |
|  | **ASV** | **Degree** | **Betweenness** | **Abundance** | **phylum** | **class** | **order** | **family** | **genus** | **species** |
| **Microbial eukaryotes** | ASV146 | 28 | 2264.1260 | 0.0432 | Dinoflagellata | Dinophyceae | Gymnodiniphycidae | Suessiaceae | Pelagodinium | unculturemarine |
|  | ASV384 | 11 | 1101.2980 | 0.0097 | Dinoflagellata | Dinophyceae | Gonyaulacales | Gonyaulacales | Pyrophacus | *Pyrophacus_steinii* |
|  | ASV281 | 13 | 965.5883 | 0.0173 | Dinoflagellata | Dinophyceae | Gonyaulacales | Gonyaulacales | Fragilidium | *Fragilidium_sp.* |
|  | ASV178 | 9 | 733.2109 | 0.0328 | Dinoflagellata | Dinophyceae | Gymnodiniphycidae | Gymnodinium_clade |  |  |
|  | ASV24 | 10 | 481.9584 | 0.3204 | Dinoflagellata | Dinophyceae | Gonyaulacales | Gonyaulacales | Pyrophacus | *Pyrophacus_steinii* |
|  | ASV67 | 10 | 450.3901 | 0.1137 | Dinoflagellata | Dinophyceae | Gonyaulacales | Gonyaulacales | Fragilidium | *Fragilidium_sp.* |
|  | ASV12 | 7 | 373.3481 | 0.7010 | Dinoflagellata | Dinophyceae | Gonyaulacales | Gonyaulacales | Alexandrium | *Alexandrium_hiranoi* |
|  | ASV10 | 4 | 300.7269 | 0.9036 | Dinoflagellata | Dinophyceae | Noctilucales | Noctilucales | Noctiluca | *Noctiluca_scintillans* |
|  | ASV339 | 3 | 123.9966 | 0.1238 | Diatomea | Bacillariophyceae | Bacillariophyceae | Bacillariophyceae | Cylindrotheca | *Cylindrotheca_closterium* |
|  | ASV114 | 5 | 78.7679 | 0.0618 | Diatomea | Diatomea | Bacillariophyceae | Bacillariophyceae | Bacillariophyceae | *Pseudo-nitzschia* |
|  | ASV272 | 4 | 61.9495 | 0.0183 | Diatomea | Coscinodiscophytina | Coscinodiscophytina | Rhizosolenids | Guinardia | *Guinardia_flaccida* |
|  | ASV135 | 2 | 33.6222 | 0.0456 | Diatomea | Bacillariophyceae | Bacillariophyceae | Bacillariophyceae |  |  |
| **Microbial prokaryotes** | ASV575 | 14 | 1740.3255 | 0.0040 | Verrucomicrobiota | Verrucomicrobiae | Arctic97B-4_marine_group | Arctic97B-4_marine_group | Arctic97B-4_marine_group | metagenome |
|  | ASV57 | 16 | 1451.1540 | 0.1483 | Alphaproteobacteria | Alphaproteobacteria | Rhodobacterales | Rhodobacteraceae |  |  |
|  | ASV13 | 21 | 1250.3161 | 0.3916 | Alphaproteobacteria | Alphaproteobacteria | Puniceispirillales | SAR116_clade | Candidatus_Puniceispirillum |  |
|  | ASV233 | 12 | 1216.6572 | 0.0227 | Alphaproteobacteria | Alphaproteobacteria | Rhodobacterales | Rhodobacteraceae |  |  |
|  | ASV48 | 17 | 1016.5868 | 0.3267 | Alphaproteobacteria | Alphaproteobacteria | Rhodobacterales | Rhodobacteraceae |  |  |
|  | ASV22 | 17 | 352.0844 | 0.1626 | Alphaproteobacteria | Alphaproteobacteria | Rhodobacterales | Rhodobacteraceae |  |  |
|  | ASV20 | 17 | 622.9975 | 0.2797 | Gammaproteobacteria | Gammaproteobacteria | KI89A_clade | KI89A_clade | KI89A_clade | obligately_oligotrophic |
|  | ASV228 | 9 | 1194.1527 | 0.0242 | Gammaproteobacteria | Gammaproteobacteria | Oceanospirillales | Halomonadaceae | Halomonas |  |
|  | ASV85 | 20 | 619.8622 | 0.1035 | Gammaproteobacteria | Gammaproteobacteria | Burkholderiales | Nitrosomonadaceae | IS-44 | uncultured_marine |
|  | ASV2 | 1 | 0.0000 | 1.6002 | Cyanobacteria | Cyanobacteria | Synechococcales | Cyanobiaceae | Synechococcus_CC9902 |  |
